# Proteomic analysis of Cry2Aa-binding proteins and their receptor function in *Spodoptera exigua*

**DOI:** 10.1038/srep40222

**Published:** 2017-01-09

**Authors:** Lin Qiu, Boyao Zhang, Lang Liu, Weihua Ma, Xiaoping Wang, Chaoliang Lei, Lizhen Chen

**Affiliations:** 1 Hubei Insect Resources Utilization and Sustainable Pest Management Key Laboratory, College of Plant Science and Technology, Huazhong Agricultural University, Wuhan 430070, People’s Republic of China

## Abstract

The bacterium *Bacillus thuringiensis* produces Crystal (Cry) proteins that are toxic to a diverse range of insects. Transgenic crops that produce Bt Cry proteins are grown worldwide because of their improved resistance to insect pests. Although Bt “pyramid” cotton that produces both Cry1A and Cry2A is predicted to be more resistant to several lepidopteran pests, including *Spodoptera exigua,* than plants that produce Cry1Ac alone, the mechanisms responsible for the toxicity of Cry2Aa in *S. exigua* are not well understood. We identified several proteins that bind Cry2Aa (polycalin, V-ATPase subunits A and B, actin, 4-hydroxybutyrate CoA-transferase [4-HB-CoAT]), and a receptor for activated protein kinase C (Rack), in *S. exigua*. Recombinant, expressed versions of these proteins were able to bind the Cry2Aa toxin *in vitro* assays. RNA interference gene knockdown of the *Se*-V-ATPase subunit B significantly decreased the susceptibility of *S. exigua* larvae to Cry2Aa, whereas knockdown of the other putative binding proteins did not. Moreover, an *in vitro* homologous competition assay demonstrated that the *Se*-V-ATPase subunit B binds specifically to the Cry2Aa toxin, suggesting that this protein acts as a functional receptor of Cry2Aa in *S. exigua*. This the first Cry2Aa toxin receptor identified in *S. exigua* brush-border membrane vesicles.

The Crystal (Cry) toxins produced by *Bacillus thuringiensis* (Bt) are a diverse group of proteins that are used to control a broad range of insect pests^1^. Not only are Bt compounds used worldwide as pesticides, but *Cry* genes have been used to create transgenic crops with enhanced resistance to pest insects. Of the Cry2A subfamily, both Cry2Aa and Cry2Ab have been successfully incorporated into plants to produce transgenic insect-resistant crops^2^,^3^.

In China, transgenic Bt cotton expressing the Cry2Ab toxin has not been commercialized. In contrast, transgenic Cry1Ac cotton, which was first cultivated in 1997, is now grown on more than 3 million hectares in 2015^4^. Adoption of this Bt cotton variety has resulted in the decline of several important pest populations at the landscape level in China, as well as reductions in the application of broad-spectrum insecticides^5^. Nonetheless, the continued large-scale planting of Bt cotton has led to new problems, including the evolution of resistance among target pests^6^,^7^ and rapid increases in non-target hemipteran^8^ and lepidopteran pests^9^,^10^,^11^. Developing plants that express more than one Cry toxin could, however, both delay insect resistance to Bt crops and increase the target pest spectrum^12^,^13^. For example, transgenic plants that express both Cry1Ac and Cry2Ab toxin would be expected to be much more resistant to lepidopteran pests, especially the beet armyworm *Spodoptera exigua*^3^,^14^.

*S. exigua* (Hübner; Lepidoptera: Noctuidae) is a polyphagous insect that has not been a significant crop pest in China for some time^11^. However, because of the recent reduction in pesticide usage in cotton fields, and because it is insensitive to Cry1Ac, the beet armyworm has once again become a major economic pest of cotton in China^3^,^15^,^16^,^17^. Although some studies suggest that *S. exigua* is less sensitive to Cry2Aa/b than to Cry1B, Cry1C or other toxins^18^,^19^, Bt crops producing both Cry1Ac and Cry2Aa/b (Cry2Ab in the case of cotton) are predicted to be more resistant to *S. exigua*, and several other lepidopteran pests, than those currently cultivated in China which produce only Cry1Ac^3^,^15^,^20^,^21^,^22^. However, except for cadherin^23^, little is known about the receptor proteins that mediate the toxicity of the Cry2A subfamily of proteins in the Lepidoptera.

In this paper, we present the first analysis of Cry2Aa receptor proteins in *S. exigua* brush-border membrane vesicles (BBMVs). Because the Cry2Aa protein has 87% sequence homology with Cry2Ab, and similar toxicity to both the Lepidoptera and Diptera, we chose Cry2Aa to represent the Cry2A subfamily^24^,^25^. In addition, and possibly more important, the purified toxin (purity > 98%) is only commercially available for Cry2Aa at present. The goal of this study was to identify Cry2Aa binding proteins in *S. exigua* BBMVs using two-dimension gel electrophoresis (2DE) and LC-MS (liquid chromatography-mass spectrometry)/MS techniques. The utility of using such a combination of protein binding assays and RNA interference to analyze the receptor function of binding proteins is also evaluated and discussed.

## Results

### Binding of Cry2Aa to *S. exigua* BBMVs

**Figure 1:**
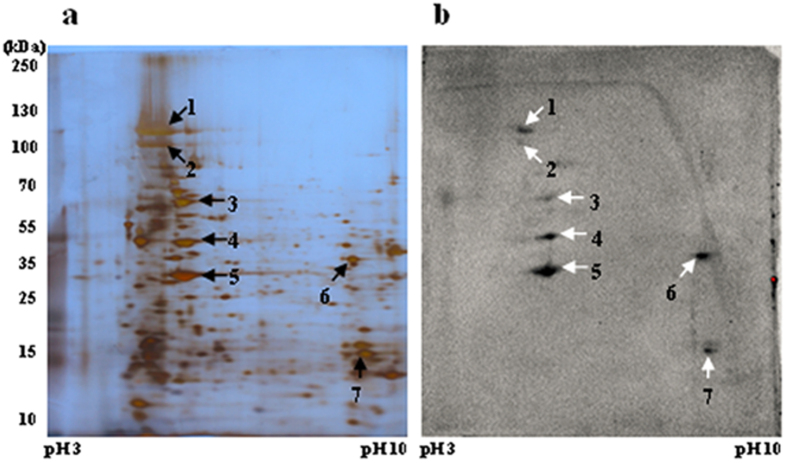
Results of 2DE analysis of solubilized *S. exigua* BBMV proteins and ligand blotting with an anti-Cry2Aa antibody. (**a**) *S. exigua* BBMV proteins (100 μg) separated by 2DE, marker positions are indicated on the left of the gel. The pH 3–10 IPG strip used for isoelectric focusing is shown at the bottom. (**b**) *S. exigua* Cry2Aa-binding proteins are the spots numbered 1 to 7; spot positions correspond to those in Fig. 1a.

Proteins of *S. exigua* BBMVs were separated by 2DE and silver stained (Fig. 1a). Proteins ranging in size from 10 kDa to 130 kDa were isolated using pH 3–10 IPG strips and 8% SDS-PAGE (sodium dodecyl sulfate-polyacrylamide gel electrophoresis) gels. Activated Cry2Aa toxin and a polyclonal antibody were used to identify specific proteins binding to Cry2Aa. An antibody-specificity test was conducted before the binding assays to confirm that the Cry2Aa antibody recognizes Cry2Aa but not Cry1Ac (Supplementary Fig. S1).

Cry2Aa bound to seven proteins of approximately 100, 110, 65, 50, 30, 35 and 15 kDa (protein spots numbered 1 through 7 in Fig. 1b). To the best of our knowledge, this is the first evidence that Cry2Aa binds to *S. exigua* BBMV proteins. Protein spots were excised from the silver-stained gel based on PVDF (polyvinylidene fluoride) membrane signals and analyzed by LC-ESI (electrospray ionization)-MS/MS. After searching protein databases, the protein spots in the silver-stained gel (Table 1) were identified as polycalin, V-type ATPase subunit A, V-type ATPase subunit B, actin, 4-hydroxybutyrate CoA-transferase (4-HB-CoAT), and a receptor for activated protein kinase C (Rack). Among these, 4-HB-CoAT and Rack were not previously known to bind to Cry toxin.

### Cloning and sequence analysis of *S. exigua* genes encoding Cry2Aa-binding proteins

We cloned the full-length of *Se*-*polycalin* cDNA (GenBank accession no. KU234093) from the midguts of *S. exigua* larvae. The 3,339-bp open reading frame (ORF) encodes a protein of 1,113 residues with a predicted mass of 122 kDa. The deduced protein sequence includes a signal peptide, glycosylphosphatidylinositol (GPI)-anchoring site, N-glycosylation sites and O-glycosylation sites (Supplementary Fig. S2). Phylogenetic analysis shows that *Se*-polycalin clusters with lepidopteran polycalin (Supplementary Fig. S3). Alignment using DNAMAN software indicates that *Se*-polycalin has highest homology with that of *Mamestra configurata* and *Helicoverpa armigera* (47.0% and 46.2%, respectively).

*S. exigua V-ATPase subunits A* and *B* were also cloned, and their sequences submitted to GenBank (KX685519 and KX685520, respectively). Their respective cDNAs contained ORFs of 1,848 and 1,482 bp, encoding 616- and 494-amino acid proteins with estimated molecular weights of 68 kDa and 55 kDa. The conserved domains walker A motif/ATP-binding site, walker B motif, N-glycosylation sites, and O-glycosylation sites, are shown in Supplementary Figs S4 and S6. Phylogenetic analysis placed both proteins in the lepidopteran clade. High identity of V-ATPase subunits A and B among diverse insect species was detected; for example, *S. exigua* V-ATPase subunit A has 95.3% identity with that of *Ostrinia furnacalis* and *Bombyx mori* and 96.3% identity with that of *Manduca sexta*, but lower identity with that of *Ceratitis capitata* (11.1%) (Supplementary Fig. S5). *S. exigua* V-ATPase subunit B also has high identity with that of *Manduca sexta* (99.2%) and *H. armigera* (99.2%) (Supplementary Fig. S7).

The sequence of *4-hydroxybutyrate CoA-transferase* was also cloned and submitted to GenBank (KX685521). The 1,431-bp cDNA obtained encodes one 52-kDa protein of 477 residues with N-glycosylation and O-glycosylation sites (Supplementary Fig. S8). It had highest identity with that of *Papilio xuthus* (87%; Supplementary Fig. S9).

### Specific expression profiles in different developmental stages and tissues

**Figure 2:**
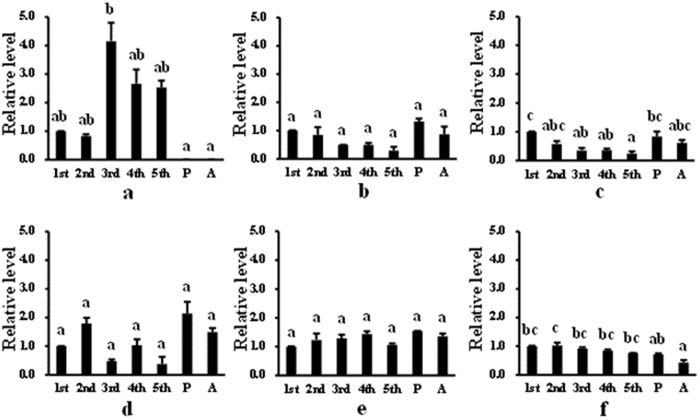
Temporal expression patterns of genes encoding putative *S. exigua* Cry2Aa-binding proteins. cDNA templates were derived from 1^st^, 2^nd^, 3^rd^, 4^th^ and 5^th^ instar larvae, pupae and adults. Three independent samples were examined for relative transcript levels using the 2^−∆∆CT^ method. a = *Se-polycalin*, b = *Se-V-ATPase subunit A*, c = *Se-V-ATPase subunit B*, d = *Se-actin*, e = *Se-4-HB-CoAT*, f = *Se*-*Rack*. Expression levels were normalized to those of the reference genes *Se*-*RpL10* and *Se*-*GAPDH*. Bars with different letters indicate *P* values < 0.05 (ANOVA).

We tested the expression patterns of all genes encoding binding proteins in different developmental stages of *S. exigua* (Fig. 2). The *Se*-*polycalin* gene was highly expressed in 3^rd^ instar larvae but was hardly expressed in pupae and adults (Fig. 2a). However, the expression of *Se*-*V-ATPase subunit A, Se*-*actin* and *Se*-*4-HB-CoAT* was not significantly different among different developmental stages (Fig. 2b,d,e). Expression of *Se*-*V-ATPase subunit B* progressively decreased from the 1st to 5th instar, and its expression in adults and 5^th^ instar larvae was moderate (Fig. 2c). Expression of the *Rack* gene was stable in different larval instars and low in adults (Fig. 2f).

**Figure 3:**
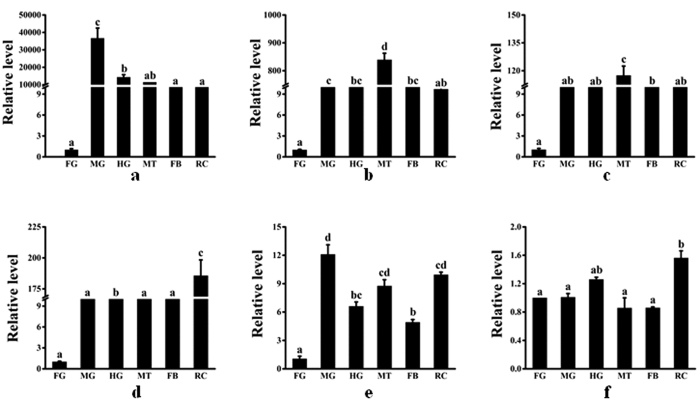
Expression of genes encoding putative *S. exigua* Cry2Aa-binding proteins in different tissues. cDNA templates were derived from the foregut (FG), midgut (MG), hindgut (HG), fat body (FB), Malpighian tubules (MT), and the remainder (R), of 4^th^ instar larvae. Three independent samples were examined for relative transcript levels using the 2^−∆∆CT^ method. a = *Se-polycalin*, b = *Se-V-ATPase subunit A*, c = *Se-V-ATPase subunit B*, d = *Se-actin*, e = *Se-4-HB-CoAT*, f = *Se-Rack*. Expression levels were normalized to those of the reference genes *Se*-*RpL10* and *Se*-*GAPDH.* Bars with different letters indicate *P* values < 0.05 (ANOVA).

Differential expression of the binding protein genes in different tissues was also measured (Fig. 3). *Se*-*polycalin* and *Se*-*4-HB-CoAT* were most highly expressed in the midgut (Fig. 3a,e) and *Se*-*V-ATPase subunit A* and *Se*-*V-ATPase subunit B* were most highly expressed in the Malpighian tubules (Fig. 3b,c). *Se*-*actin* and *Se*-*Rack* were most highly expressed in the remaining tissues (Fig. 3d,f).

### RNA interference knockdown of binding proteins

**Figure 4:**
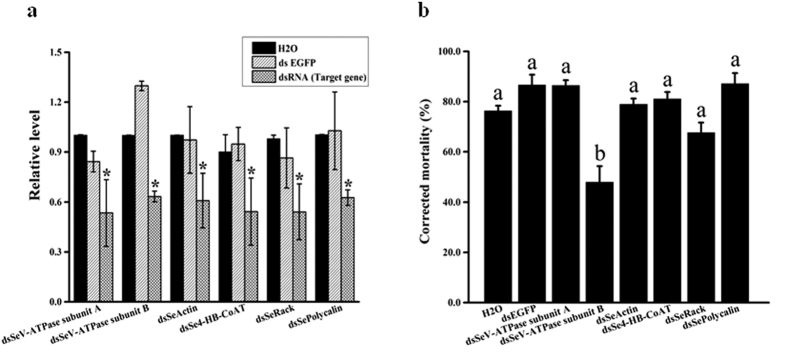
RNA interference knockdown of transcripts of putative *S. exigua* Cry2Aa-binding proteins in *S. exigua* larvae and subsequent susceptibility of larvae to Cry2Aa toxin. (**a**) Relative expression of target gene transcript levels in *S. exigua* neonate larvae that had been allowed to feed for 48 h on an artificial diet to which either water, EGFP dsRNA, or *Se*-target genes dsRNA, had been added. Relative estimates of target gene transcript levels were obtained by qRT-PCR and normalized to those of *RpL10* and *GAPDH*. *P < 0.05 (ANOVA). (**b**) Corrected mortality of different treatment groups after feeding on a diet containing 2.6 μg/cm^2^ Cry2Aa toxin. The corrected mortality is based on 5 replicates. Bars with different letters indicate *P* values < 0.05 (ANOVA).

Compared to dsEGFP or H_2_O, larval ingestion of dsRNAs specific for the *Se*-V-ATPase subunit A, *Se*-V-ATPase subunit B, *Se*-actin, *Se*-4-HB-CoAT, *Se*-Rack, and *Se*-polycalin, significantly reduced transcript levels of these genes by 46.6%, 36.7%, 39.1%, 45.8%, 45.9% and 37.4%, respectively (Fig. 4a). Corrected mortalities following ingestion of Cry2Aa toxin for each of the above dsRNA treatment groups were 86.4%, 47.9%, 78.9%, 81.1%, 67.7% and 87.1%, respectively. The mortality of larvae fed dsRNA specific for *Se*-V-ATPase subunit B was significantly lower than that of the water or dsEGFP control groups (Fig. 4b).

### Production of recombinant peptides and binding assays

**Figure 5:**
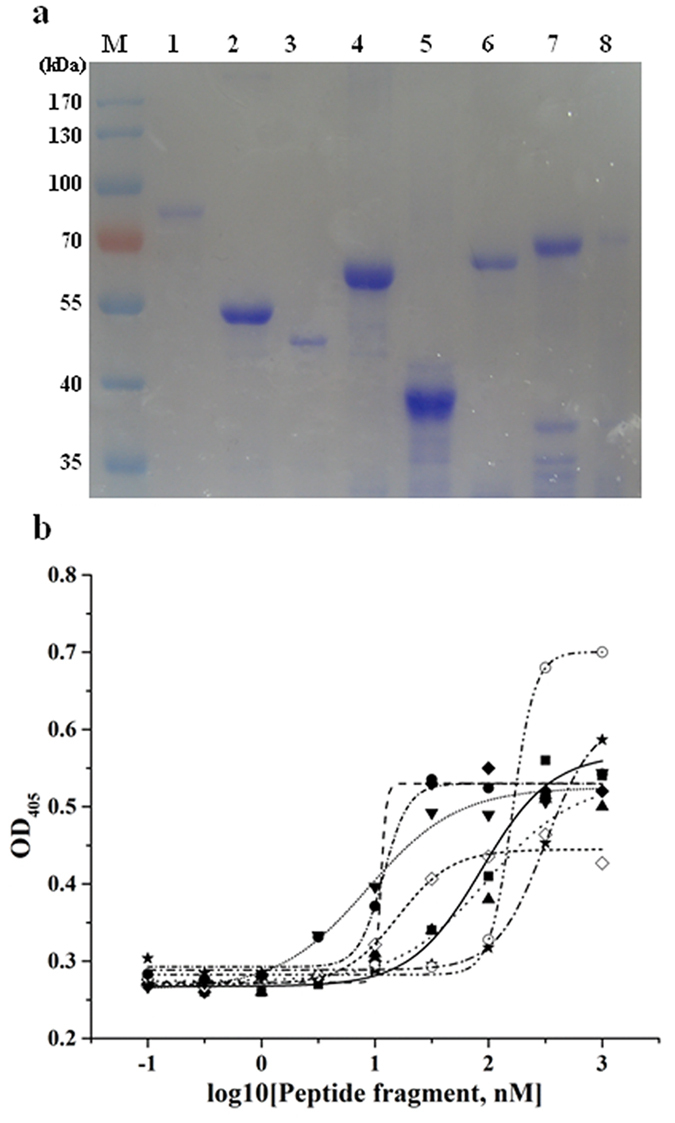
Binding of Cry2Aa to recombinant peptides. (**a**) Positions of purified, recombinant peptides after staining with Coomassie Blue on SDS–PAGE gel. Peptides had been bacterially expressed and purified in a nickel-nitrilotriacetic acid (Ni-NTA) affinity column. Lanes 1 to 8 are: the *Se*-V-ATPase subunit A, *Se*-V-ATPase subunit B, *Se*-actin, *Se*-4-HB-CoAT, *Se*-Rack, and three truncated recombinant *Se*-polycalin peptides (6 = peptide1, 7 = peptide2, 8 = peptide3). (**b**) Degree of binding, as indicated by optical density (OD), of Cry2Aa to different *Se*-peptide fragments. *Se*-V-ATPase subunit A (■), *Se*-V-ATPase subunit B (♦), *Se*-actin (▲), *Se*-4-HB-CoAT (★), *Se*-Rack (○), *Se*-polycalin peptide1 (◇), *Se*-polycalin peptide2 (▼) and *Se*-polycalin peptide3 (●).

Expressed peptides were purified and separated by 8% SDS-PAGE gels (Fig. 5a). The results of an ELISA (enzyme-linked immunosorbent assay) indicate that the *Se*-V-ATPase subunit A, *Se*-V-ATPase subunit B, *Se*-actin, *Se*-4-HB-CoAT, *Se*-Rack and three partial fragments of *Se*-polycalin, all bound to Cry2Aa toxin (Fig. 5b).

### Dot blot analysis of the Cry2Aa receptor in *S. exigua*

**Figure 6:**
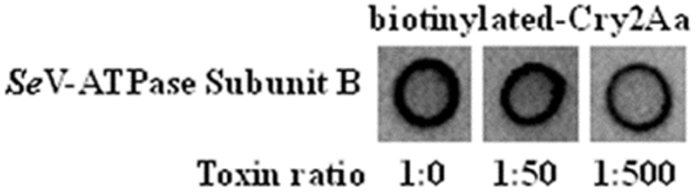
Results of homologous competitive binding assay of recombinant *Se*-V-ATPase subunit B peptide to Cry2Aa toxin. Weight ratios of 1:0, 1:50 and 1:500 of unlabeled Cry2Aa toxin were used to compete with the *Se*-V-ATPase subunit B in binding to biotinylated-Cry2Aa.

Based on the previous bioassays, we conducted homologous, competitive binding assays to test the specificity of binding between Cry2Aa and the recombinant *Se*-V-ATPase subunit B peptide. Binding between Cry2Aa and the *Se*-V-ATPase subunit B peptide was markedly reduced at higher concentrations of un-labelled Cry2Aa (Fig. 6).

## Discussion

Our results indicate that *S. exigua* V-ATPase subunit B is associated with Cry2Aa toxicity. Identifying this novel putative Cry2Aa receptor is potentially crucial to understanding how Cry2Aa is toxic to lepidopteran species. Since V-ATPase was first reported in *Saccharomyces cerevisiae*^26^, a substantial amount of evidence has demonstrated that V-ATPases, which are located in the goblet cell apical membrane and rely on ATP hydrolysis to actively pump their substrates across membranes, are involved in energy production and conversion^27^,^28^. V-ATPase in the insect midgut mediates pH to create an alkaline environment and participates in ion-transport processes^29^. V-ATPase up-regulation has been found to be related to Cry1Ac resistance in *Plodia interpunctella* and *P. xylostella*^30^,^31^. Although V-ATPase has been identified as a Cry toxin-binding protein in *H. virescens* (Cry1Ac), *H. armigera* (Cry1Ac) and *O. furnacalis* (Cry1Ab)^32^,^33^,^34^, little is known about its function with regard to Cry toxins in other insects. Interestingly, RNA silencing of the *A. aegypti* ATP synthase subunit beta increased larval mortality to Cry toxins, which suggests that this protein is involved in Cry toxin resistance^35^.

The model proposed by Jurat-Fuentes *et al*.^36^ postulates that the Cry toxin binds to cadherin and is then inserted into the cell membrane, facilitating interaction of the toxin with other molecules such as V-ATPase. In fact, Cry1Ac binds to V-ATPase and disturbs H^+^/K^+^ transport, thereby destabilizing pH^37^. Moreover, Cry1Ac has been found to inhibit (Na^+^, K^+^)-ATPase in mammals^38^. Two main classes of active transporters comprise the active transmembrane transport system. Sodium solute symporter (SSS) is driven by proton or sodium transmembrane gradients^39^, and has been shown to act as a Cry toxin receptor^40^. Secondary active transporters include ATP-binding cassette (ABC) proteins and V-ATPase, both of which are ATP-dependent electrogenic proton pumps that actively move substrates across cell membranes. ABC proteins are also involved in Cry toxicity^41^,^42^,^43^ and our results suggest that V-ATPase interacts with Cry2Aa toxin. However, further research is required to determine whether the V-ATPase subunit B interacts with Cry toxin in a manner similar to that of the ABC transporter.

Our results show that *Se*-*Polycalin* was most highly expressed in the midgut of the 3^rd^ to 5^th^ larval instars of *S. exigua*, which may protect the larval midgut from viruses or oxidative damage^44^. *Se*-*V-ATPase subunit B* was most highly expressed in 1^st^ instar larvae and adults, and *Se*-*V-ATPase subunit A, Se*-*V-ATPase subunit B*, and *Se*-*4*-*HB-CoAT*, were most highly expressed in the gut and Malpighian tubules, which suggests that they may be involved in energy metabolism^27^,^28^,^45^. *Se*-*Actin* and *Se*-*Rack* showed high transcript levels in other larval tissues, perhaps because their proteins comprise part of the cytoskeleton^46^. We also performed *in vitro* binding assays which showed that the polycalin peptides 2 and 3 had the highest binding affinities with Cry2Aa, followed by V-ATPase subunit B. These different binding affinities may have occurred because different binding proteins have different binding sites for the Cry toxin^47^.

Although cadherin is a crucial receptor in lepidopterans, including *S. exigua*^23^, we did not identify cadherin as a receptor in this study. This could be because LC-MS technology is relatively insensitive to proteins that are not abundant; we found that cadherin was not abundant in a previous study. Another possible explanation is that 2DE technology does not effectively resolve proteins of high molecular weight; *Se-*cadherin has a molecular weight of approximately 200 kDa^48^.

Although we identified seven putative Cry2Aa-binding midgut proteins, the results of our RNAi knockdown experiment suggest that only the *Se*-V-ATPase subunit B is a functional receptor for Cry2Aa in *S. exigua*.

## Materials and Methods

### Insect rearing and BBMV preparation

*S. exigua* larvae were collected from the campus greenhouse of Huazhong Agricultural University in June 2012 and reared on an artificial diet according to the protocol described by Qiu *et al*.^23^. *S. exigua* BBMVs were prepared from fourth-instar larvae according to the method described by Wolfersberger *et al*.^49^, with minor modifications. Protein concentrations were determined using the method described by Bradford (1976)^50^ with bovine serum albumin (BSA) as the standard.

### 2DE and ligand blotting

Proteins were extracted and precipitated with a ReadyPrep^TM^ 2-D Clean-up Kit (BIO-RAD). Precipitated proteins were dissolved in solubilization buffer (7 M urea, 2 M thiourea, 4% CHAPS, 65 mM dithiothreitol (DTT), 0.2% (w/v) Bio-Lyte, 0.001% bromophenol blue). For 2D electrophoresis, Readystrip^TM^ IPG strips (11 cm long, pH 3–10, nonlinear; BIO-RAD) were rehydrated overnight with solubilized protein. Following rehydration, the strips were subjected to isoelectric focusing using a PROTEAN IEF CELL following the manufacturer’s recommendations (BIO-RAD). The focused strips were equilibrated for 12 min in equilibration buffer (6 M urea, 2% SDS, 20% glycerol, 0.375 M Tris-HCl [pH 8.8]) containing 1% DTT followed by a second equilibration for 12 min in equilibration buffer plus 4% iodoacetamide. For 2D separation, the strips were overlaid on 8% SDS-PAGE gels for electrophoresis. The separated proteins were either stained or transferred to polyvinylidene difluoride (PVDF) membranes.

After proteins had been transferred to PVDF membranes, the membranes were blocked in PBST buffer (135 mM NaCl, 2 mM KCl, 10 mM Na_2_HPO_4_, 1.7 mM KH_2_PO_4_, 0.1% Tween-20, pH 7.5) containing 5% (w/v) skim milk for 2 h, then incubated with 0.3 μg/ml activated Cry2Aa (EnviroLogix Inc., Portland, ME, USA) in blocking buffer for 2 h at room temperature. The membranes were washed in PBST buffer three times, then incubated for 2 h with a polyclonal antibody against Cry2Aa (diluted 1:3,500; Genscript Biology Company, Nanjing, China). After washing as above, the membranes were incubated with a goat anti-rabbit IgG horseradish peroxidase (HRP)-linked antibody (diluted 1:5,000). After final washes, the membranes were developed with an ECL chemiluminescence detection kit (Fermentas/Thermo Fisher Scientific, Waltham, MA, USA) following the manufacturer’s recommendations.

### Mass spectrometry

After blotting, areas on the gel were excised according to the PVDF membrane signals and destained with destaining solution (30% acetonitrile/100 mM NH_4_HCO_3_). Each gel sample was then subject to a series of processes, including incubation with 100 mM DTT at 56 °C for 30 minutes, treatment with 200 mM indole-3-acetic acid (IAA) after removal of the supernatant, and incubation with 100 mM NH_4_HCO_3_. The liquid was removed, and 100% acetonitrile was added for 5 minutes. The samples were freeze-dried before being subject to trypsin digestion for 24 hours at 37 °C, then analyzed using liquid chromatography-electrospray ionization tandem mass spectrometry (LC-ESI-MS/MS) at the Shanghai Life Science Research Institute, Chinese Academy of Sciences, Shanghai, China. The amino acid sequence results were compared with those in the NCBI database using Mascot2.2 software.

### Gene cloning and sequence analysis

Total RNA was isolated from the midguts of actively feeding 4^th^ instar *S. exigua* larvae using the RNAiso reagent (TaKaRa, Dalian, China) after contaminating genomic DNA had first been eliminated with RNase-free DNase. The RNA preparation was subject to reverse transcription with the PrimeScript^TM^ RT reagent Kit (TaKaRa, China), according to the manufacturer’s instructions. Partial *Se*-*polycalin, Se*-*V-ATPase subunit A, Se*-*V-ATPase subunit B* and *Se*-*4-hydroxybutyrate CoA-transferase* cDNA sequences were obtained from Prof. Fei Li (Zhejiang University). A smart RACE (rapid amplification of cDNA ends) cDNA amplification kit (Clontech, TaKaRa Bio Inc., Dalian, China) was used to amplify full-length target genes from *S. exigua* larvae for which pairs of gene-specific primers were designed using Primer 5.0 software based on the partial sequences (Supplementary Tables S1–S5). The PCR products were subcloned into the PMD (18)-T vector (Takara, Dalian, China) and sequenced by the Nanjing Genscript Company, China. The resultant sequences were submitted to GenBank.

Full-length cDNAs were subjected to bioinformatic analysis using an ORF finder tool (http://www.ncbi.nlm.nih.gov/gorf/gorf.html). Sequence alignment was performed using DNAMAN software, and phylogenetic analysis was performed using MEGA4.0^51^,^52^. Amino acid sequences from other species were used to construct a phylogenetic tree (Supplementary Table S8). Deduced protein sequences were obtained using the ExPASy translate tool Translate (http://web.expasy.org/translate/) from the Swiss Institute of Bioinformatics. N-terminal signal peptides were predicted using the SignalP 4.0 server (http://www.cbs.dtu.dk/services/SignalP/). The GPI modification site prediction server (big-PI Predictor: http://mendel.imp.ac.at/sat/gpi/gpi_server.html was used to predict GPI-anchor signal sequences and GPI anchoring sites. The presence of N- and O-glycosylation sites in predicted protein sequences was assessed using NetNGlyc 1.0 (http://www.cbs.dtu.dk/services/NetNGlyc/) and NetOGlyc 4.0 (http://www.cbs.dtu.dk/services/NetOGlyc/), respectively.

### Production of recombinant *S. exigua* proteins and microtiter plate binding assays

A method similar to a recently described protocol^23^ was used to produce recombinant proteins. Briefly, PCR products were purified with Wizard PCR Preps DNA Purification System (Promega, Madison, WI, USA) and double digested with FastDigest restriction enzymes (Fermentas, Thermo Fisher Scientific, USA) for 10 min at 37 °C. The products were ligated into the previously digested pET-30a (+) vector to generate pET-30a/*Se*-peptide plasmids. However, we failed to obtain a *Se*-polycalin fragment using this expression system, and the pGEX-6P-1 vector was therefore used to construct the recombinant plasmid pGEX-6P-1/*Se*-polycalin containing a His tag (see primers in Supplementary Table S1). *Se*-polycalin was divided into three fragments corresponding to bases 1–1,113, 1,114–2,226 and 2,227–3,339 of the *Se*-polycalin coding sequence, which were named peptide1, peptide2 and peptide3. Insert sequence and orientation were confirmed by sequencing by the Genscript Biology Company, Nanjing, China.

For expression, 200 ng of each recombinant plasmid was transformed into *Escherichia coli* strain BL21 (DE3) (TransGen Biotech, Inc, Beijing, China) and positive clones were cultured overnight at 37 °C in LB medium containing 50 mg/ml kanamycin. When the absorbance was 0.5 to 0.8 at 600 nm, protein expression was induced with 0.1 mM isopropyl-D-thiogalactoside (IPTG) for 6 h at 37 °C, with horizontal shaking at 200 rpm. The *E. coli* cells were harvested by centrifugation at 10,000× g for 10 min at 4 °C, after which pellets were resuspended in a solution of 20 mM sodium phosphate, 500 mM sodium chloride, 30 mM imidazole and 5 M urea (pH 7.4), containing 1 mM phenylmethanesulfonyl fluoride (PMSF). Cells were lysed by sonication for 15 min on ice. The protein fragments with a His tag were purified using a nickel-nitrilotriacetic acid (Ni-NTA) affinity column (HisTrap HP column, GE Healthcare Life Sciences, Piscataway, NJ, USA) and eluted with eluting buffer (20 mM sodium phosphate, 500 mM sodium chloride, 500 mM imidazole and 5 M urea, pH 7.4). The cleaved proteins were refolded by dialysis using a gradient of decreasing urea concentration. The purified proteins were analyzed using SDS-PAGE gels, and protein concentrations determined using the Bradford^50^ method described above.

An ELISA for measuring the binding of *Se*-polycalin, *Se*-V-ATPase subunit A, *Se*-V-ATPase subunit B, *Se*-actin, *Se*-Rack, and *Se*-4-HB-CoAT, peptides to Cry2Aa in microplates was developed using a protocol described by Chen *et al*.^53^. In brief, 96-well plates containing 0.5 μg Cry2Aa per well were incubated for 12 h at 4 °C, followed by three washes with PBST buffer and treatment with blocking buffer (PBS, 0.1% Tween 20, 0.5% gelatine) for 1 h at 37 °C. The ELISA plates were washed, then incubated with 0–1,000 nM *S. exigua* recombinant peptides for 2 h at 37 °C. After washing, an anti-His antibody (1:5,000) (Genscript Biology Company, Nanjing, China) was added and incubated overnight at 4 °C. After additional washes, followed by incubation with a secondary goat-anti-mouse-alkaline phosphatase (1:2,000) antibody for 2 h at 37 °C, freshly prepared substrate (3 mM nitrophenyl phosphate) was added. The reaction was stopped with 0.02 M NaOH, and the absorbance at 405 nm was measured using a Bio-Rad microplate reader.

### Competitive binding dot blot assay

Activated Cry2Aa protein was biotinylated using the EZ-Link sulpho-N-hydroxysuccinimide (NHS) liquid chromatography (LC) biotinylation kit (Pierce, FL, USA) according to the manufacturer’s instructions. A homologous competitive binding assay for Cry2Aa by *S. exigua* recombinant peptides was then conducted. In total, 2 μg of purified peptides bound to a nitrocellulose membrane was blocked in PBST containing 3% BSA and incubated for 3 h with 0.2 μg/ml biotinylated Cry2Aa and unlabelled toxin at weight ratios of 1:0, 1:50 and 1:500. Following washing, streptavidin-HRP was used to detect biotinylated toxin using an ECL chemiluminescence detection kit (Fermentas/Thermo Fisher Scientific, Waltham, MA USA), as described previously^23^.

### RNA interference knockdown of *S. exigua* target genes

A method adapted from Ren *et al*.^54^ was used to produce a dsRNA-expressing vector. Target gene fragments were amplified from *S. exigua* midgut cDNA using PrimeSTAR HS DNA polymerase (TaKaRa Bio Inc., Dalian, China). The products were individually cloned into the plasmid pET-2P to generate recombinant pET2P/*Se*-target-gene plasmids. pET2P/*EGFP* recombinant plasmid production of EGFP dsRNA was used to generate the control EGFP dsRNA^23^. Recombinant plasmids were transferred into competent *E. coli* HT115 (DE3) cells. Individual colonies were cultured at 37 °C in 500 ml LB medium containing 50 μg/ml kanamycin. After reaching an OD600 of 1.0, the production of dsRNA was induced by the addition of 0.4 mM IPTG, and the cultures were allowed to grow for an additional 5 h at 37 °C. The bacteria were precipitated by centrifugation at 5,000 rpm for 10 min, and dsRNA was extracted according to the method described by Timmons *et al*.^55^ and Dong *et al*.^56^. Nucleic acids were analyzed for appropriate size by 1% agarose gel electrophoresis.

Newly hatched larvae were allowed to feed for 48 h at 27 °C on an artificial diet to which either 50 μg/cm^2^ *Se*-target genes, EGFP dsRNA, or water, had been added. The larvae were then transferred to the wells of a 6-well plate where they were allowed to continue feeding for 7 days at 27 °C. Each well contained 5 ml of artificial diet plus either 2.6 μg/cm^2^ activated Cry2Aa toxin (equivalent to the LC_70_ value determined by a pilot study), or water (the control). Five replicates were conducted with a total of 120 larvae in each treatment.

### qPCR assay

Three groups of samples were prepared, including different tissues, developmental stages and dsRNA treatment groups (three replicates for each treatment). Quantitative real-time PCR (qPCR) was used to measure differences in gene expression between tissues, developmental stages and dsRNA treatment groups. qPCR primers were designed using the NCBI profile server (http://www.ncbi.nlm.nih.gov/tools/primer-blast) (Supplementary Tables S1–S5). *S. exigua RpL10* and *GAPDH* were used as internal reference genes (Supplementary Table S6)^54^,^57^. The following standard qPCR protocol was used: denaturation at 95 °C for 30 s, followed by 40 cycles of 95 °C for 10 s and 59 °C for 30 s. All qPCR samples were run in triplicate using SYBR Premix ExTaq™ (TaKaRa) and a Bio-Rad Detection iQ2 System. Melting curve analysis from 55 to 95 °C was performed to determine the specificity of the qPCR primers. To determine the efficiency of qRT-PCR primers, a 5-fold dilution series of second-instar larval cDNA corresponding to 1 μg total RNA was used to produce a standard curve (cDNA concentration vs. Ct) with efficiencies calculated from the slope using linear regression. The corresponding qRT-PCR efficiencies were calculated according to the equation: E = (10^[1/slope]^ − 1)*100^58^,^59^ (Supplementary Table S7).

### Data analysis

Quantitative expression data were analyzed using the 2^−∆∆Ct^ method^58^. Corrected larval mortalities were calculated using Abbott’s formula^60^. Means and variances of treatments were analyzed with one-way ANOVA implemented in SPSS for Windows (SPSS 18.0, Chicago, IL, USA).

## Additional Information

**How to cite this article**: Qiu, L. *et al*. Proteomic analysis of Cry2Aa-binding proteins and their receptor function in *Spodoptera exigua. Sci. Rep.* **7**, 40222; doi: 10.1038/srep40222 (2017).

**Publisher's note:** Springer Nature remains neutral with regard to jurisdictional claims in published maps and institutional affiliations.

## Supplementary Material

Supplementary Information

## Figures and Tables

**Table 1:** Summary of Cry2Aa-binding proteins identified in *S. exigua* BBMVs based on the NCBI database and using Mascot2.2 software.

dot^a^	Accession No^b^	MW(kDa)	PI	Name	Species
1	gi|327082384	32.7	4.48	polycalin	*Trichoplusia ni*
2	gi|327082384	32.7	4.48	polycalin	*Trichoplusia ni*
3	gi|401323	68.46	5.14	V-type ATPase subunit A	*Nasonia vitripennis*
4	gi|401326	55.1	5.26	V-type ATPase subunit B	*Helicoverpa armigera*
5	gi|157111829	41.9	5.29	Actin	*Aedes aegypti*
6	gi|389613607	51.1	8.33	4-hydroxybutyrate CoA-transferase	*Papilio xuthus*
7	gi|328670883	36.4	7.64	Receptor for activated protein kinase C	*Helicoverpa armigera*

^a^Numbers correspond to those in Fig. 1.

^b^Proteins in the NCBI database for which significant peptide mass matches or sequence similarity were available.
