# Supplementary material for: Proteomic analysis of Cry2Aa-binding proteins and their receptor function in Spodoptera exigua
Source: Sci Rep. 2017 Jan 9;7:40222. doi: 10.1038/srep40222 (PMC5220377; doi:10.1038/srep40222)
Supplement: Supplementary Information [file srep40222-s1.pdf]

SUPPLEMENTARY INFORMATION

Proteomic analysis of Cry2Aa-binding proteins and their receptor function in *Spodoptera exigua*

Lin Qiu, Boyao Zhang, Lang Liu, Weihua Ma, Xiaoping Wang, Chaoliang Lei & Lizhen Chen*

Hubei Insect Resources Utilization and Sustainable Pest Management Key Laboratory, College of Plant Science and Technology,

Huazhong Agricultural University, Wuhan 430070, People's Republic of China.

* Corresponding author: Lizhen Chen

Email: lzchen@mail.hzau.edu.cn

Tel: +86-27-87287207

Fax: +86-27-87287207

Supplementary Figures

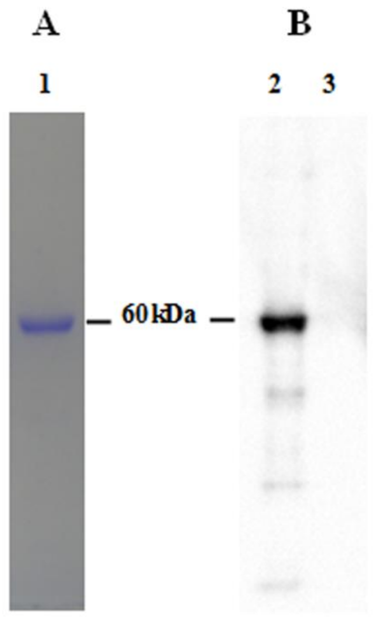

Figure S1. Results of a Western blot test of Cry2Aa polyclonal antibody specificity. (A) Band produced by 10 μg Cry2Aa toxin on 8% SDS-PAGE after staining with Coomassie Blue (line 1). (B) Banding produced by Cry2Aa antibody in the presence of Cry2Aa (line 2) and Cry1Ac (line 3) after proteins had been transferred to PVDF membrane. Presence of band in line 2 and absence of band in line 3 shows that the Cry2Aa antibody is specific for Cry2Aa and not Cry1Ac.

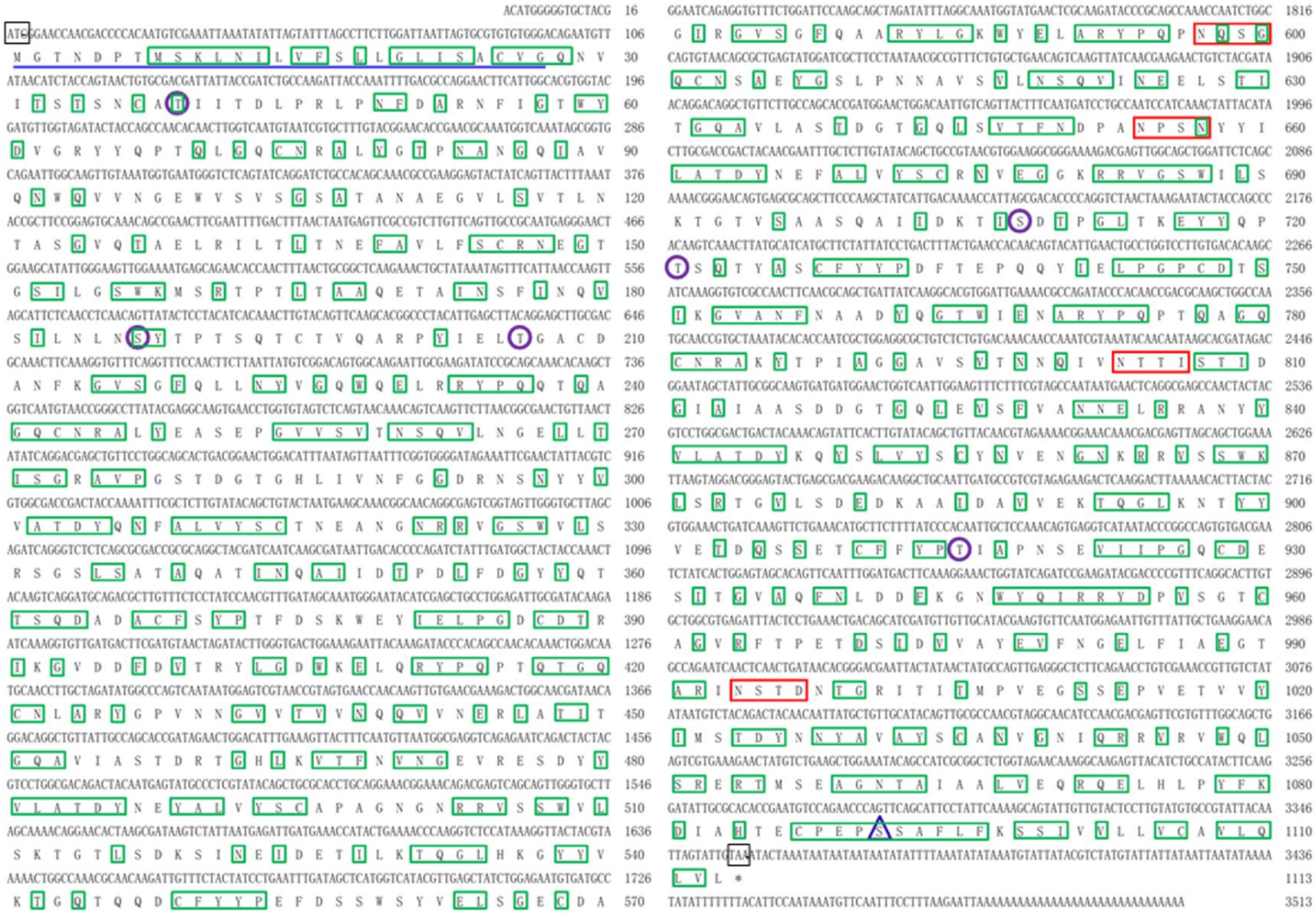

Figure S2. Amino acid sequence and domain structure of *Spodoptera exigua* polycalin (GenBank accession no. AEA76321.1). The start (ATG) and stop (TAA) codons are in black boxes. The signal peptide (SP) is underlined in blue and the GPI-anchoring site is marked with a blue triangle. Positions of the four predicted N-glycosylation sites are indicated by red boxes and putative O-glycosylation sites are circled in purple. Conserved amino acid residues are in green boxes.

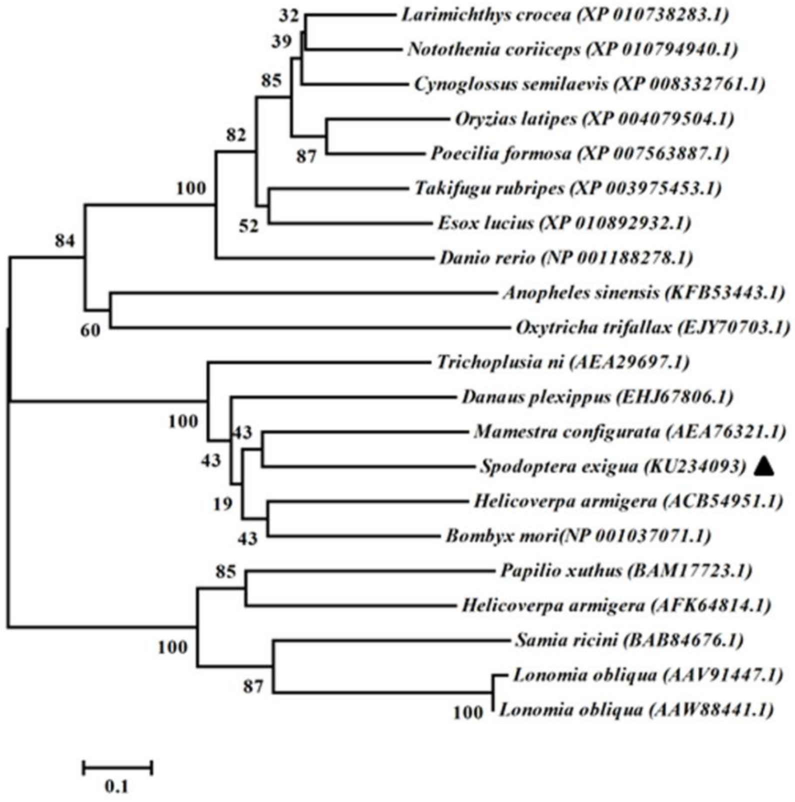

Figure S3. A neighbor-joining (NJ) consensus tree of the polycalin amino acid sequences constructed using MEGA 4.0. Bootstrap values are expressed as percentages of 500 replications and shown at the main branch points. GenBank accession numbers of genes from different species are shown in parentheses. The position of the *Spodoptera exigua* polycalin gene is shown by a black triangle.

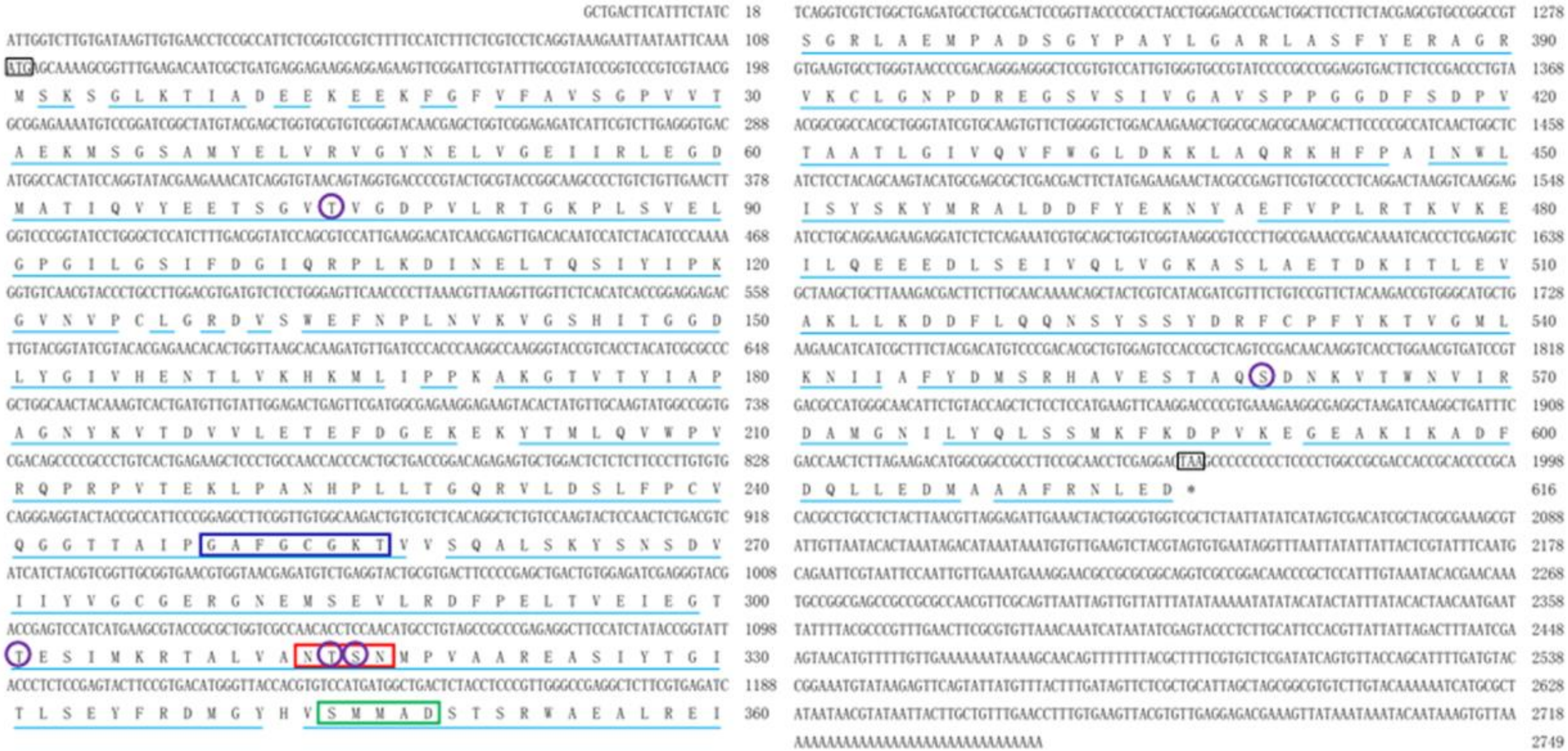

Figure S4. Multiple sequence alignment of *Spodoptera exigua* V-ATPase subunit A (GenBank accession no. P31400.1). Numbers on the right indicate nucleotide (upper) and amino acid (lower) positions. The start (ATG) and stop (TAA) codons are in black boxes. The red box indicates the N-glycosylation site, putative O-glycosylation sites are circled in purple. The location of the Walker A motif/ATP binding site is indicated by the purple box and that of the conserved Walker B motif by the green box. There are no SP and GPI-anchoring sites. Conserved fragments are underlined in light-green.

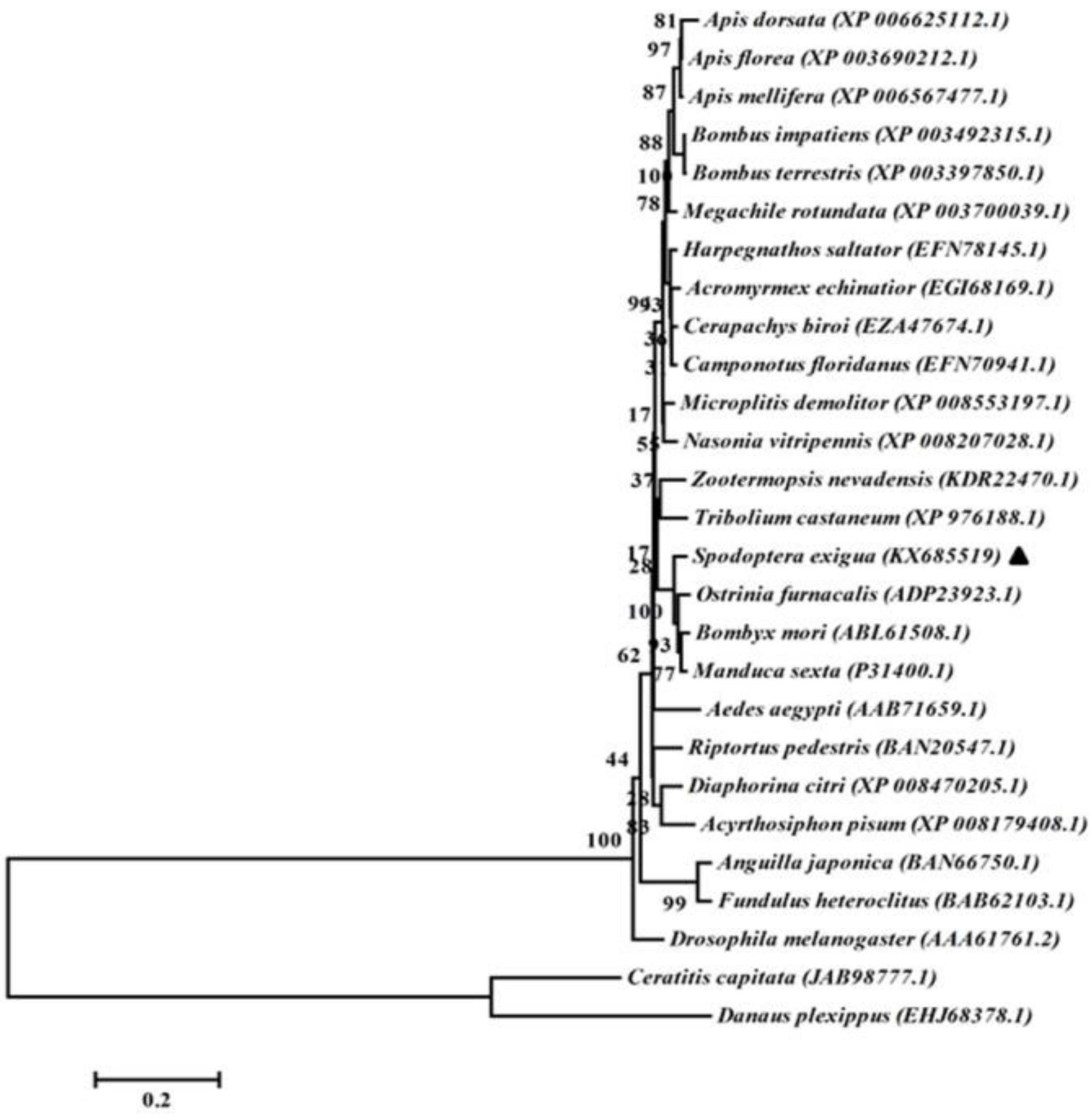
81
97
87
88
100
78
99
93
36
3
17
55
37
17
28
100
93
62
77
44
28
83
100
99
Apis dorsata (XP 006625112.1)
Apis florea (XP 003690212.1)
Apis mellifera (XP 006567477.1)
Bombus impatiens (XP 003492315.1)
Bombus terrestris (XP 003397850.1)
Megachile rotundata (XP 003700039.1)
Harpegnathos saltator (EFN78145.1)
Acromyrmex echinatior (EGI68169.1)
Cerapachys biroi (EZA47674.1)
Camponotus floridanus (EFN70941.1)
Microplitis demolitor (XP 008553197.1)
Nasonia vitripennis (XP 008207028.1)
Zootermopsis nevadensis (KDR22470.1)
Tribolium castaneum (XP 976188.1)
Spodoptera exigua (KX685519) ▲
Ostrinia furnacalis (ADP23923.1)
Bombyx mori (ABL61508.1)
Manduca sexta (P31400.1)
Aedes aegypti (AAB71659.1)
Riptortus pedestris (BAN20547.1)
Diaphorina citri (XP 008470205.1)
Acyrthosiphon pisum (XP 008179408.1)
Anguilla japonica (BAN66750.1)
Fundulus heteroclitus (BAB62103.1)
Drosophila melanogaster (AAA61761.2)
Ceratitis capitata (JAB98777.1)
Danaus plexippus (EHJ68378.1)
0.2

Figure S5. Phylogenetic tree of insect V-ATPase subunit A amino acid sequences constructed using MEGA 4.0. Bootstrap values are expressed as percentages of 500 replications and shown at the main branch points. GenBank accession numbers of genes from different species are shown in parentheses. The position of the *Spodoptera exigua* V-ATPase subunit A gene is shown by a black triangle.

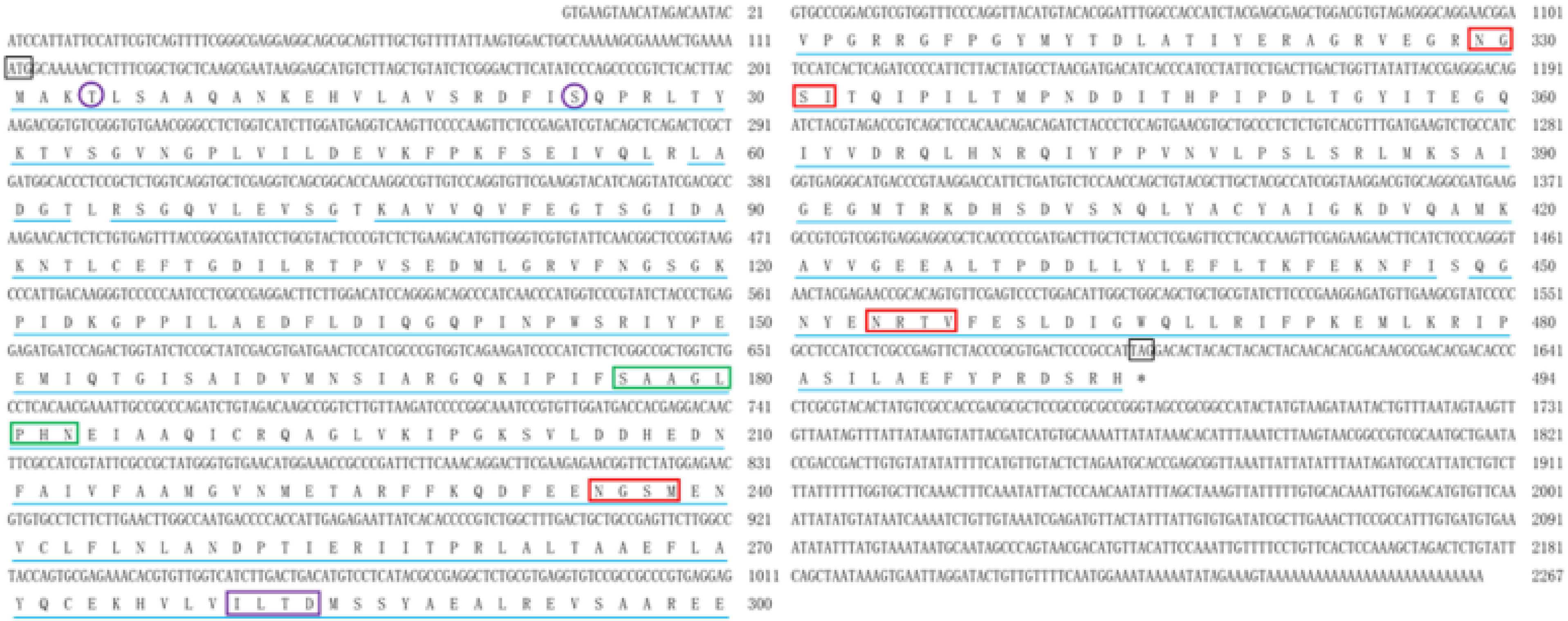

Figure S6. Structural features of the full-length of the *Spodoptera exigua* V-ATPase subunit B (GenBank accession no. P31401.1). The start (ATG) and stop (TAG) codons are in black boxes. N-glycosylation sites are in red boxes and putative O-glycosylation sites are circled in purple. The location of the Walker B motif is indicated by the purple box and that of the homologous, conserved Walker A motif by the green box. There are no SP and GPI-anchoring sites. The conserved fragment is underlined in light-green.

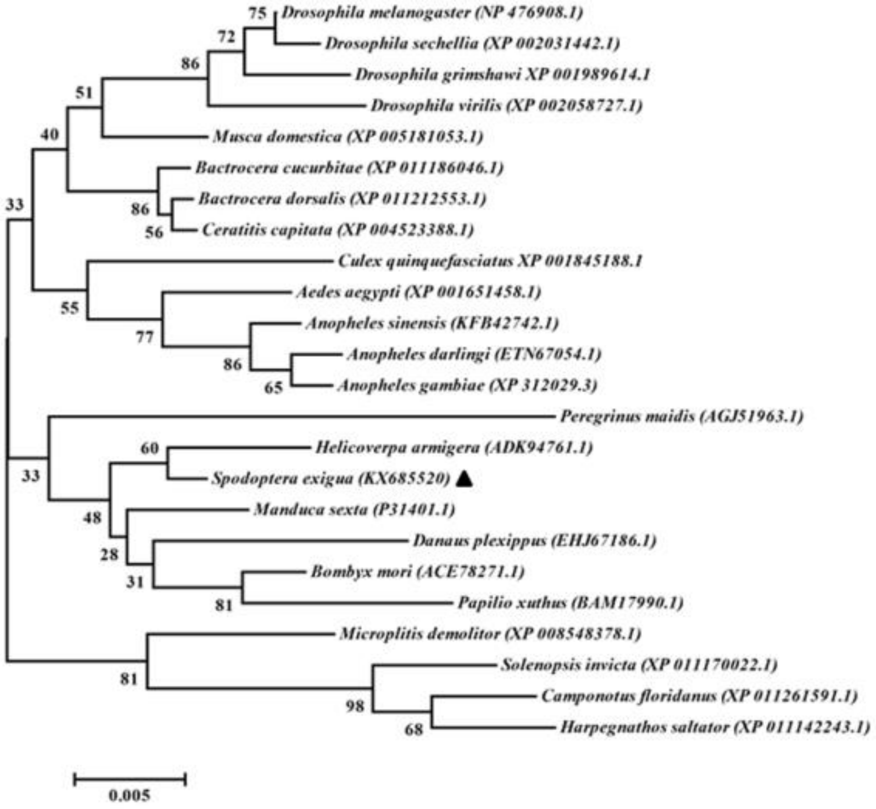

Figure S7. Phylogenetic tree of insect V-ATPase subunit B amino acid sequences constructed using MEGA 4.0. Bootstrap values are expressed as percentages of 500 replications and shown at the main branch points. GenBank accession numbers of genes from different species are shown in parentheses. The position of the *Spodoptera exigua* V-ATPase subunit B gene is shown by a black triangle.

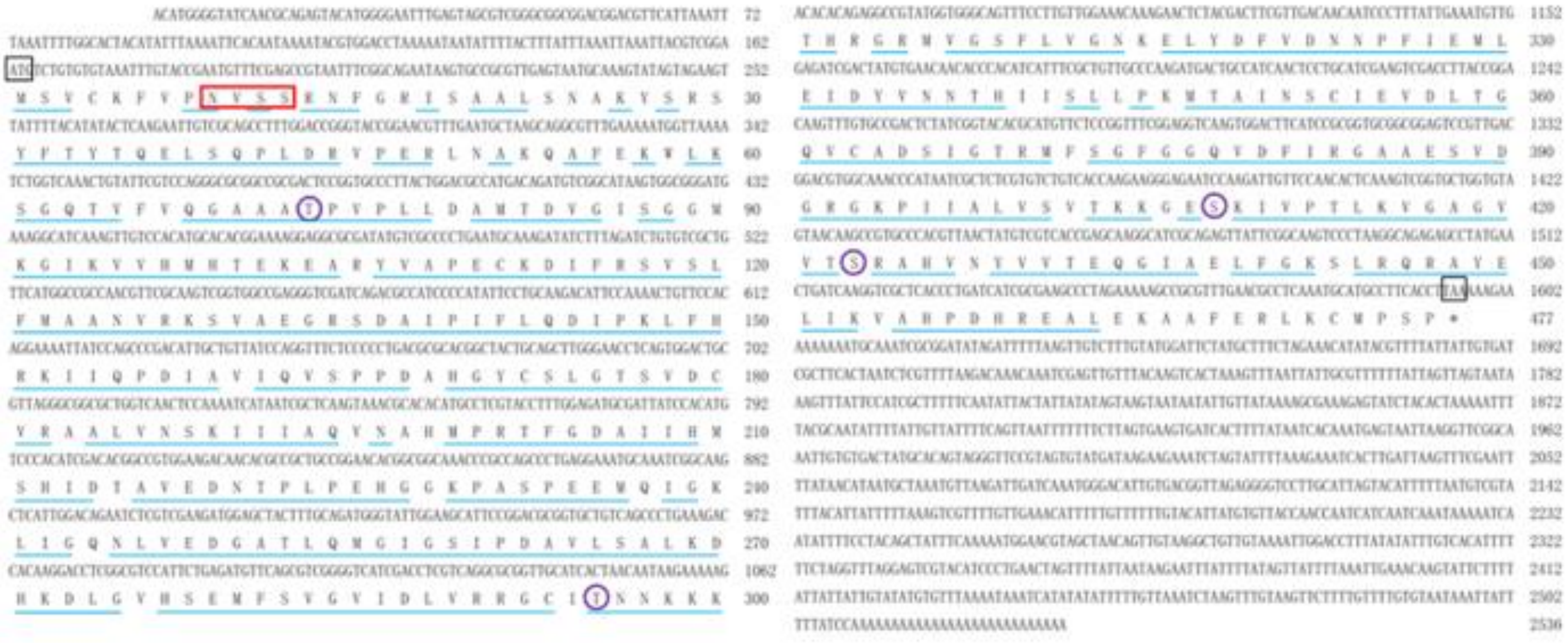

Figure S8. Amino acid sequence and domain structure of *Spodoptera exigua* 4-HB-CoAT (GenBank accession no. BAM20135.1). The start (ATG) and stop (TAA) codons are highlighted in black boxes. The location of the N-glycosylation site is indicated by the red box and putative O-glycosylation sites are circled in purple. Conserved amino acid residues are underlined in light-green.

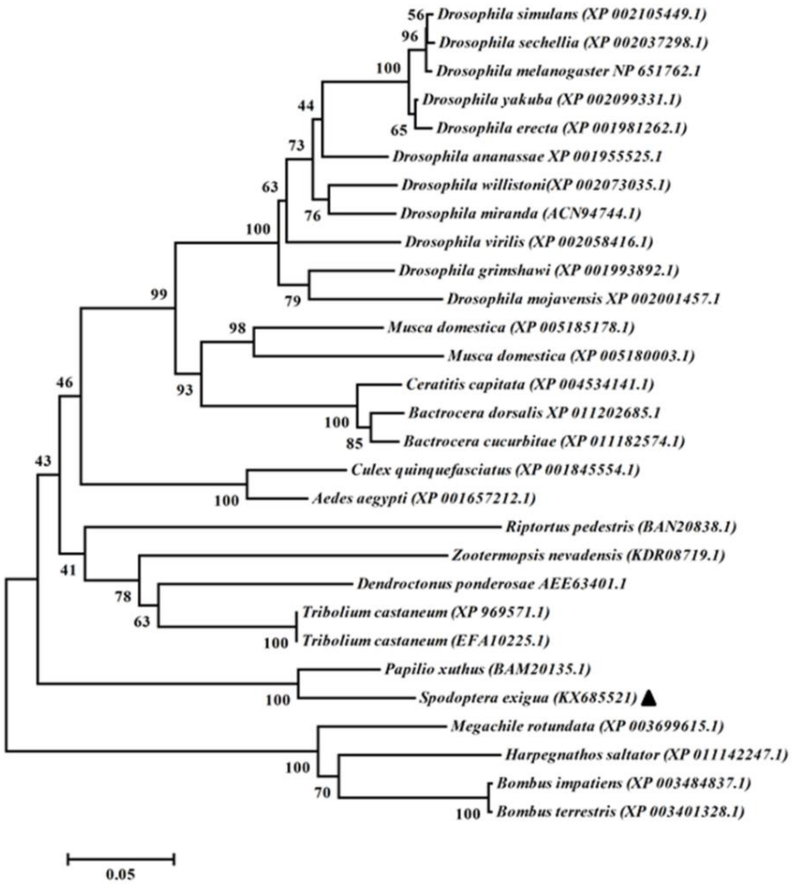

Figure S9. Phylogenetic tree of insect 4-hydroxybutyrate CoA-transferase (4-HB-CoAT) amino acid sequences constructed using MEGA 4.0. Bootstrap values are expressed as percentages of 500 replications and shown at the main branch points. GenBank accession numbers of genes from different species are shown in parentheses.

Supplementary Tables

Table S1

Nucleotide primers used to clone full-length *S. exigua* polycalin cDNA by 5'- and 3'- RACE, and those used for protein expression, dsRNA synthesis and qRT-PCR.

**Primer**	**Primer Sequence (5'–3')**
**5'RACE**	
*Se*Polycalin 5GSP	CATTTGCGTTCGGTGTTCCGTATGTAGC
*Se*Polycalin 5NGSP	TGGCAGATCGGTAATAATCGTCGCACAGT
3'RACE	
*Se*Polycalin 3GSP	CGCTGTCTCTGTGACAAACAACCAAATCG
*Se*Polycalin 3NGSP	AGTGATGATGGAACTGGTCAATTGGAAGT
**Primers for protein expression**	
Peptide 1F	atGGATCCCATCACCACCATCACCATCTGCCTGGTCCTTGTGACACAA
Peptide 1R	atCTCGAGATGGTGATGGTGATGGTGCAATACTAATTGTAATACGGCA
Peptide 2F	atGGATCCCATCACCACCATCACCATCCAACGTTTGATAGCAAATGGGA
Peptide 2R	atCTCGAGATGGTGATGGTGATGGTGTTCAATGTACTGTTGTGGTTCA
Peptide 3F	atGGATCCCATCACCACCATCACCATATGGGAACCAACGACCCCACAA

Peptide 3R	atCTCGAGATGGTGATGGTGATGGTGATAGGAGAAACAAGCGTCTGCA
**Primers for dsRNA Synthesizing**	
ds*Se*Policalin-F	gcGAATTCTCGAATTTTGACTTTAACTAA
ds*Se*Policalin-F	gtcGAATTCATTTCTATCCCCACCGAAATTAA
**Primers for qRT-PCR**	
*Se*Policalin qPCR-F	TCTGCCACAGCAAACGCCGA
*Se*Policalin qPCR-R	GCGGCAACTGAACAAGACGGC

Table S2

Nucleotide primers used to clone full-length *S. exigua* V-ATPase subunit A cDNA by 5'-and 3'-RACE, and those used for protein expression, dsRNA synthesis and qRT-PCR.

**Primer**	**Primer Sequence (5'–3')**
**5'RACE**	
*Se*V-ATPase subunit A 5GSP	AGTCGGCAGGCATCTCAGCCAGACG
*Se*V-ATPase subunit A 5NGSP	GCTCGTTGTACCCGACACGCACCAG
**3'RACE**	
*Se*V-ATPase subunit A 3GSP	GTTAGGAGATTGAAACTACTGGCGT
*Se*V-ATPase subunit A 3NGSP	TGTTTACTTTGATAGTTCTCGCTGC
**Primers for protein expression**	
*Se*V-ATPase subunit A-F	tgGGATCCATGAGCAAAAGCGGTTTGAAGACA
*Se*V-ATPase subunit A-R	atGTCGACcGTCCTCGAGGTTGCGGAAGGC
**Primers for dsRNA Synthesizing**	
ds*Se*V-ATPase subunit A -F	tgGAATTCAGACGACTTCTTGCAACAAAACAGC
ds*Se*V-ATPase subunit A -F	atgcGAATTCCGACCTCCGCGCGGCGTTCCTT
**Primers for qRT-PCR**	

*Se*V-ATPase subunit A qPCR-F	TTTGACGGTATCCAGCGTCC
*Se*V-ATPase subunit A qPCR-R	GAGACATCACGTCCAAGGCA

Table S3

Nucleotide primers used to clone full-length *S. exigua* V-ATPase subunit B cDNA by 5'-and 3'-RACE, and those used for protein expression, dsRNA synthesis and qRT-PCR.

**Primer**	**Primer Sequence (5'–3')**
**5'RACE**	
*Se*V-ATPase subunit B 5GSP	TGGGGGACCCTTGTCAATGGGCTTAC
*Se*V-ATPase subunit B 5NGSP	ACCAGAGCGGAGGGTGCCATCAGCGA
**3'RACE**	
*Se*V-ATPase subunit B 3GSP	TAAGGACCATTCTGATGTCTCCAACC
*Se*V-ATPase subunit B 3NGSP	TCCCAGGGTAACTACGAGAACCGCAC
**Primers for protein expression**	
*Se*V-ATPase subunit B-F	acGAATTCATGGCAAAAACTCTTTCGGCTGCT
*Se*V-ATPase subunit B-R	atCTCGAGCATGGCGGGAGTCACGCGGGTAGA
**Primers for dsRNA Synthesizing**	
ds*Se*V-ATPase subunit B -F	atGAATTCGTAGCCGCGGCCATACTATGT
ds*Se*V-ATPase subunit B -F	cgGAATTCGAATACAGAGTCTAGCTTTGG
**Primers for qRT-PCR**	
*Se*V-ATPase subunit B qPCR-F	ACAGCTCAGACTCGCTGATG
*Se*V-ATPase subunit B qPCR-R	AGAGAGTGTTCTTGGCGTCG

Table S4

Nucleotide primers used to clone full-length *S. exigua* 4-HB-CoAT cDNA by 5’-and 3’-RACE, and those used for protein expression, dsRNA synthesis and qRT-PCR.

Primer	Primer Sequence (5’–3’)
**5’RACE**	
*Se*4-HB-CoAT 5GSP	AACAAGGAAACTGCCCACCATACGG
*Se*4-HB-CoAT 5NGSP	CGCCGAGGTCCTTGTGGTCTTTCAG
**3’RACE**	
*Se*4-HB-CoAT 3GSP	GTCGGCATAAGTGGCGGGATGAAAGG
*Se*4-HB-CoAT 3NGSP	GCTCTCGTGTCTGTCACCAAGAAGGG
**Primers for protein expression**	
*Se*4-HB-CoAT -F	gcGGATCCATGTCTGTGTGTAAATTTGTACCG
*Se*4-HB-CoAT -R	atCTCGAGTAGGTGAAGGCATGCATTTGA
**Primers for dsRNA Synthesizing**	
ds*Se*4-HB-CoAT -F	atGAATTCTCCGATCTCAATCAAATTCA
ds*Se*4-HB-CoAT F	atGAATTCGATCGACCCTCGGCCACCG
**Primers for qRT-PCR**	
*Se*4-HB-CoAT qPCR-F	ATTCGGCAAGTCCCTAAGGC
*Se*4-HB-CoAT qPCR-R	GCATGCATTTGAGGCGTTCA

Table S5

Nucleotide primers used to clone *Spodoptera exigua* activated C kinase 1 receptor (Rack) and actin cDNA, and those used for protein expression, dsRNA synthesis and qRT-PCR.

Primer	Primer Sequence (5'–3')
**Primers for protein expression**	
*Se*Rack -F	ATggatccATGACTGAAACATTGAAGCTTAGA
*Se*Rack -R	ATgtcgacTTCGTGCTGAGACTGACACCTGC
*Se*Actin -F	tgGAATTCATGTGCGACGACGATGTTGCTGCGCT
*Se*Actin -R	cgAAGCTTGAAGCACTTGCGGTGGACGATGCCG
**Primers for dsRNA Synthesizing**	
ds*Se*Rack -F	ATgaattcTGTGTGCGTTTCTCCCCCAACCA
ds*Se*Rack- R	ATgaattcTCCTCACAGTCAGGATACGTAAGT
ds*Se*Actin-F	tgGAATTCAAGGAGAAGCTGTGCTATGTCGCCC
ds*Se*Actin-F	gcGAATTCTGCACGCTTAGAAGCACTTGCGGTGGA
**Primers for qRT-PCR**	
*Se*Rack qPCR-F	GGGCTGGAGTGTTGGTGTTA
*Se*Rack qPCR-R	TGTCGGGGTATTTCGGGTTG
*Se*Actin qPCR-F	GCGACGACGATGTTGCTGCG
*Se*Actin qPCR-R	ACGAGGGCGACCTACGATGGA

Table S6

Nucleotide primers used to perform qRT-PCR on *Spodoptera exigua* glyceraldehyde-3-phosphate dehydrogenase (GAPDH) and ribosomal protein L10 (RpL10).

Primer	Primer Sequence (5'–3')
SeGAPDH qPCR-F	CTGAGGAACAGGTCGTGTCA
SeGAPDH qPCR-R	TTCAGAGAGATACCGGCAGCA
Se RpL10 qPCR-F	CTCTGCGTCGTGCCAAGTTC
Se RpL10 qPCR-R	CCTCACGCAGCTTCTCGAAT

Table S7

Specifications for optimized qRT-PCR amplification of *Spodoptera exigua* Polycalin, V-ATPase subunit A, V-ATPase subunit B, Actin, 4-hydroxybutyrate CoA-transferase (4-HB-CoAT), receptor for activated protein kinase C (Rack), GAPDH and RpL10.

**Gene name**	**Efficiency**	**R2**	**Slope**
SeRpL10	101.1%	0.996	-3.296
SeGAPDH	100.3%	0.999	-3.316
SePolycalin	100.0%	0.998	-3.322
SeV-ATPase A	100.2%	0.999	-3.316
SeV-ATPase B	100.0%	0.999	-3.322
Se4-HB-CoAT	100.0%	0.996	-3.322
SeRack	100.2%	0.997	-3.317
SeActin	92.4%	0.992	-3.520

Table S8
Amino acid sequences of Cry2Aa binding proteins used to construct phylogenetic trees.

Code name	Organism	Accession number
**Polycalin**	*Mamestra configurata*	AEA76321.1
	Helicoverpa armigera	ACB54951.1
	Bombyx mori	NP_001037071.1)
	Danaus plexippus	EHJ67806.1
	Trichoplusia ni	AEA29697.1
	Anopheles sinensis	KFB53443.1
	Oxytricha trifallax	EJY70703.1
	Danio rerio	NP_001188278.1
	Papilio xuthus	BAM17723.1
	Cynoglossus semilaevis	XP_008332761.1
	Oryzias latipes	XP_004079504.1
	Larimichthys crocea	XP_010738283.1
	Helicoverpa armigera	AFK64814.1
	Poecilia formosa	XP_007563887.1
	Lonomia obliqua	AAV91447.1
	Takifugu rubripes	XP_003975453.1
	Samia ricini	BAB84676.1
	Esox lucius	XP_010892932.1
	Notothenia coriiceps	XP_010794940.1
	Lonomia obliqua	AAW88441.1
**V-ATPase subunit A**	*Drosophila melanogaster*	AAA61761.2
	Aedes aegypti	AAB71659.1

	Ostrinia furnacalis	ADP23923.1
	Bombyx mori	ABL61508.1
	Microplitis demolitor	XP_008553197.1
	Diaphorina citri	XP_008470205.1
	Zootermopsis nevadensis	KDR22470.1
	Nasonia vitripennis	XP_008207028.1
	Acyrthosiphon pisum	XP_008179408.1
	Tribolium castaneum	XP_976188.1
	Cerapachys biroi	EZA47674.1
	Ceratitis capitata	JAB98777.1
	Apis dorsata	XP_006625112.1
	Apis mellifera	XP_006567477.1
	Riptortus pedestris	BAN20547.1
	Acromyrmex echinatior	EGI68169.1
	Harpegnathos saltator	EFN78145.1
	Camponotus floridanus	EFN70941.1
	Megachile rotundata	XP_003700039.1
	Apis florea	XP_003690212.1
	Danaus plexippus	EHJ68378.1
	Bombus impatiens	XP_003492315.1
	Bombus terrestris	XP_003397850.1
	Anguilla japonica	BAN66750.1
	Fundulus heteroclitus	BAB62103.1
	Manduca sexta	P31400.1
**V-ATPase subunit B**	*Manduca sexta*	P31401.1
	Helicoverpa armigera	ADK94761.1

	Danaus plexippus	EHJ67186.1
	Bombyx mori	ACE78271.1
	Bactrocera dorsalis	XP_011212553.1
	Bactrocera cucurbitae	XP_011186046.1)
	Ceratitis capitata	XP_004523388.1
	Musca domestica	XP_005181053.1
	Papilio xuthus	BAM17990.1
	Anopheles sinensis	KFB42742.1
	Drosophila melanogaster	NP_476908.1
	Anopheles darlingi	ETN67054.1
	Drosophila sechellia	XP_002031442.1
	Microplitis demolitor	XP_008548378.1
	Drosophila virilis	XP_002058727.1
	Aedes aegypti	XP_001651458.1
	Drosophila grimshawi	XP_001989614.1）
	Culex quinquefasciatus	XP_001845188.1
	Solenopsis invicta	XP_011170022.1
	Peregrinus maidis	AGJ51963.1
	Camponotus floridanus	XP_011261591.1
	Harpegnathos saltator	XP_011142243.1
	Anopheles gambiae	XP_312029.3
**4-HB-CoAT**	*Papilio xuthus*	BAM20135.1
	Culex quinquefasciatus	XP_001845554.1
	Riptortus pedestris	BAN20838.1
	Aedes aegypti	XP_001657212.1
	Dendroctonus ponderosae	AEE63401.1

Tribolium castaneum	XP_969571.1
Drosophila virilis	XP_002058416.1
Drosophila yakuba	XP_002099331.1
Drosophila erecta	XP_001981262.1
Drosophila willistoni	XP_002073035.1
Drosophila miranda	ACN94744.1
Musca domestica	XP_005185178.1
Drosophila simulans	XP_002105449.1
Drosophila melanogaster	NP_651762.1
Drosophila ananassae	XP_001955525.1）
Tribolium castaneum	EFA10225.1
Ceratitis capitata	XP_004534141.1
Drosophila grimshawi	XP_001993892.1
Drosophila mojavensis	XP_002001457.1
Bactrocera dorsalis	XP_011202685.1
Bactrocera cucurbitae	XP_011182574.1
Zootermopsis nevadensis	KDR08719.1
Musca domestica	XP_005180003.1
Bombus impatiens	XP_003484837.1
Bombus terrestris	XP_003401328.1
Megachile rotundata	XP_003699615.1
Harpegnathos saltator	XP_011142247.1
Drosophila sechellia	XP_002037298.1
